# Introduction to Special Abstract Section on Disseminating New Knowledge and Practices for Asian and Pacific Islanders

**DOI:** 10.31372/20200503.1112

**Published:** 2020

We are very pleased to publish our first special section of student and community abstracts and brief reports. Students of all disciplines and community members interested in disseminating new knowledge and practices submitted their papers. By accepting abstracts as well as short papers, a new opportunity was afforded for students, faculty, and community members to share their scholarship relative to the health of Asian/Pacific Islanders. The *Asian/Pacific Island Nursing Journal* is pleased to support and promote their scholarly endeavors particularly from those of Asian/Pacific Islander background.

Congratulations to S. Robert Spence Jr., a DNP-FNP student at Washington State University, who was awarded the first student scholarship for publication in the *APIN* student/community papers special issue. He is the first author for the abstract “Gaining Entrée into a Micronesian Islander-Based Community Organization Through Culturally Responsive Team Building and Reflection.”

Special Section Editors

May Kealoha, PhD, MSN, MPH

Jillian Inouye, PhD, FAAN

